# Rapid, automated detection of stem canker symptoms in woody perennials using artificial neural network analysis

**DOI:** 10.1186/s13007-015-0100-8

**Published:** 2015-12-24

**Authors:** Bo Li, Michelle T. Hulin, Philip Brain, John W. Mansfield, Robert W. Jackson, Richard J. Harrison

**Affiliations:** East Malling Research, New Road, East Malling, ME19 6BJ Kent, UK; Faculty of Natural Sciences, Imperial College London, SW7 2AZ London, UK; School of Biological Sciences, University of Reading, Reading, RG6 6AJ UK

**Keywords:** Stem canker, Artificial neural network, Image analysis

## Abstract

**Background:**

*Pseudomonas syringae* can cause stem necrosis and canker in a wide range of woody species including cherry, plum, peach, horse chestnut and ash. The detection and quantification of lesion progression over time in woody tissues is a key trait for breeders to select upon for resistance.

**Results:**

In this study a general, rapid and reliable approach to lesion quantification using image recognition and an artificial neural network model was developed. This was applied to screen both the virulence of a range of *P. syringae* pathovars and the resistance of a set of cherry and plum accessions to bacterial canker. The method developed was more objective than scoring by eye and allowed the detection of putatively resistant plant material for further study.

**Conclusions:**

Automated image analysis will facilitate rapid screening of material for resistance to bacterial and other phytopathogens, allowing more efficient selection and quantification of resistance responses.

**Electronic supplementary material:**

The online version of this article (doi:10.1186/s13007-015-0100-8) contains supplementary material, which is available to authorized users.

## Background

The bacterial phytopathogen *Pseudomonas syringae* encompasses pathovars that infect over 180 plant species. Three distinct clades of *P. syringae* (pv *morsprunorum* race 1, pv. *morsprunorum* race 2 and pv. *syringae*) are the major causal agents of bacterial canker of *Prunus* species grown worldwide [1]. This genus of stone fruit trees includes economically important species such as cherry and plum. The bacteria are able to infect all aerial plant organs, including leaves, blossom and fruit. Severe damage to the tree occurs when bacteria infect woody tissues via wounds or leaf scars to produce necrotic cankers that are often associated with extensive gummosis [2]. These cankers cause girdling of branches and may result in dieback or eventual death of the tree when affecting the main trunk [3]. The disease commonly results in tree losses of approximately 20 %, however, in severe cases, losses of up to 75 % have been reported in the US [4, 5].

Current control methods for this disease are limited. They include good hygiene when pruning, to reduce the likelihood of infection and the use of copper-based spays to control epiphytic bacterial populations [6]. The breeding of resistant cultivars, complemented with excellent sanitation methods, would be the most effective control of this disease [7]. At present, no cultivars have been shown to exhibit complete resistance; however there is variation in disease susceptibility [2], meaning breeding approaches could be successful. Therefore, a rapid disease screening method would be highly beneficial in *Prunus* breeding programmes, to allow the identification of resistant genotypes.

Susceptibility to bacterial canker is usually determined by visually assessing natural infection in the field over several years [8]. This approach is time consuming and different environmental conditions between fields may lead to misleading results [9]. Several rapid laboratory-based assays have been proposed, including the use of cut shoots [3, 8, 10], immature fruits [11, 12] and micro-propagated plantlets [9] to examine disease susceptibility. In this study we assessed the use of the cut shoot assay to screen *Prunus* cultivars for susceptibility to bacterial canker. The assay involves inoculating first-year dormant shoots with *P. syringae* and estimating disease severity based on the extent of necrosis. This approach, although more rapid than field-based observations, was found to be variable between assessors, being based on a subjective appraisal of lesion development and therefore lacked reproducibility, as has been shown in other similar studies [13]. A more rapid and high-throughput alternative to visual assessment involves the use of automated image analysis software [14, 15].

Automated image analysis is becoming a popular tool for plant disease assessment as it potentially provides greater speed, accuracy and reliability [16]. Nilsson [17] was the first to report the utility of remote sensing and image analysis for plant pathology. After Nilsson, various studies successfully applied image analysis in the visible region for disease severity assessment [18–22], with such techniques excellently reviewed in [23]. Digital image analysis has been compared with visual disease assessment for several diseases such as coffee rust [24], powdery mildew [25], yellow rust [26] and citrus canker [27]. These studies indicated that colour or monochrome image analysis provided more accurate measurement, whilst drastically reducing the time required for examination [16, 28].

Among the different image analysis algorithms used to measure disease severity, the conversion from RGB (Red Green Blue) to HSI (Hue, Saturation and Intensity) colour space is commonly used and the hue value has been considered to be an effective channel to discriminate healthy and diseased areas on colour images [16]. The hue channel threshold can be set manually or automatically to segment diseased from healthy areas using software such as Adobe Photoshop [29], ASSESS© [30], Scion image software (Scion Corporation, Frederick, MD) [21], ImageJ [31] or other custom developed software programs [32, 33].

Other more sophisticated algorithms have been proposed for the automatic classification of plant diseases using colour images. Naikwadi [34] converted RGB images to HSI format and applied Spatial Gray-level Dependence Matrices (SGDM) as the colour co-occurrence texture analysis method for only H (hue) and S (saturation) images. Grey-level co-occurrence methodology was used to calculate the features, which were inputted into neural networks for recognition. Apart from HSI colour space, colour images have also been converted to the L1 L2 L3 colour model for disease area measurement [18, 35]. Schikora [19] utilised this method for the image-based analysis of plant infection with human pathogens. The L2 and L3 values plus the information of the surrounding pixels were classified via supervised learning techniques such as neural networks or support vector machines.

The use of Artificial Neural Networks (ANN) has recently become a popular tool of pattern recognition in image analysis [36] and disease quantification [37]. ANN is an efficient computational model inspired by the parallel nervous systems of animals [38]. It is widely implemented in machine learning and has been applied to the food and agricultural industry [39, 40]. The use of ANN has also been trialed for detection and quantification of various plant diseases [41–44]. The whole system is based upon an interconnection of neurons, which computes the output from the input variables. Besides input and output layers, ANN systems always have one or more hidden layers between them. A training dataset is used to update the adaptive weights of all the neurons in order to minimize the mean square error between the output and ideal values below a certain criteria [38].

This paper reports the development of an automated image analysis software which utilises ANN to analyse images of cherry and plum shoots exhibiting necrosis due to bacterial canker, with the goal of improving the accuracy of disease resistance screening. The software developed reduces the time and subjectivity involved in disease assessment and has the potential to be applied during screening of other important tree diseases.

## Results and discussion

### Quantification based on automated image analysis

A feed-forward artificial neural network (ANN), which is also known as multi-layer perceptrons (MLP), was implemented for the classification of diseased and healthy shoot tissue (see “Methods” section for full details). The recognition of diseased area is based on the colour, and only R, G and B values were used as the input variables of the ANN model. The training samples consisted of pixels labelled as healthy and diseased, and in total 75,155 pixels were manually labeled from 13 images, covering all the variation in colour due to disease. All the images were taken under the same illumination, and the colours of the diseased region showed little variation. The image analysis was applied to 420 images of inoculated shoots, producing estimates of percentage area and length of necrosis to determine disease severity.

**Fig. 1:**
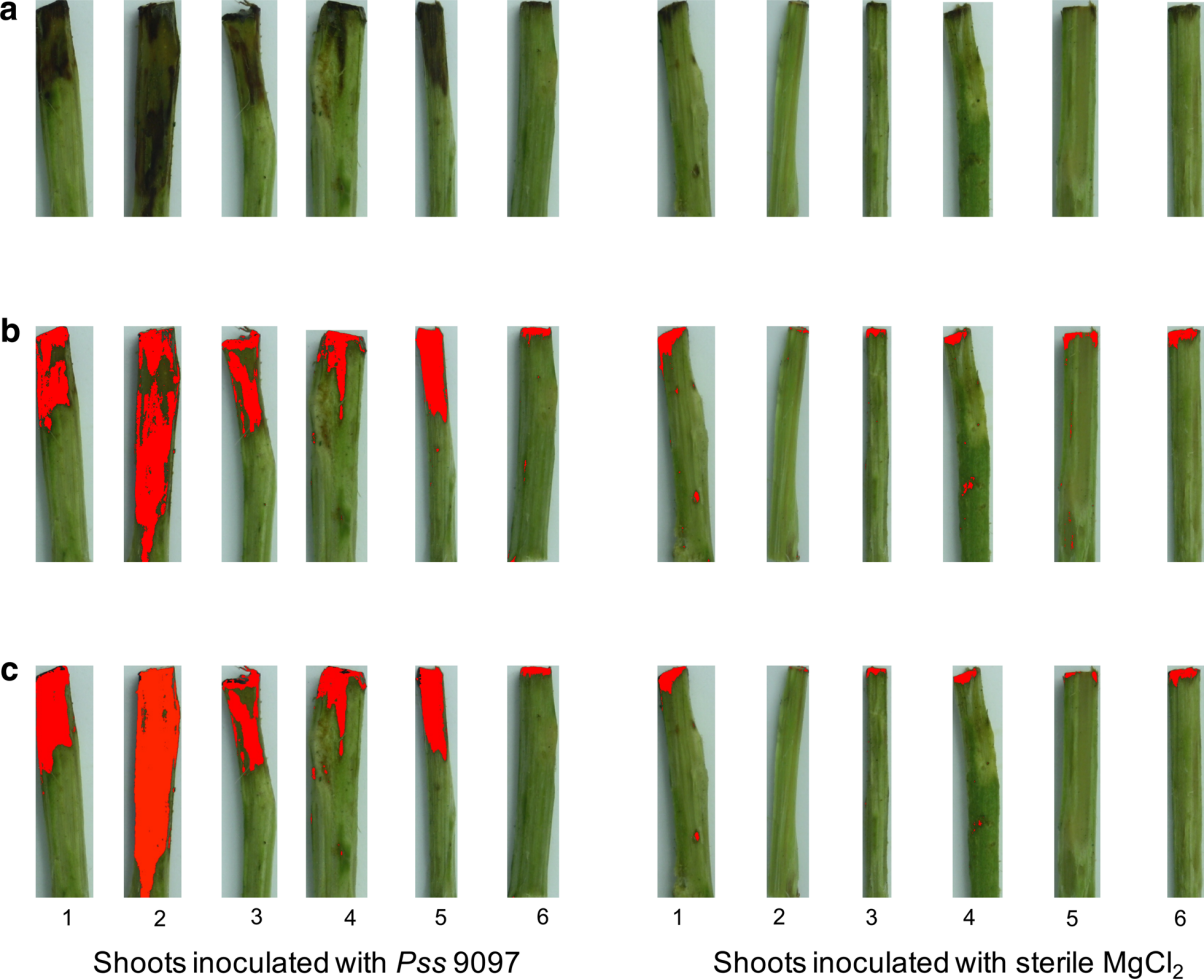
Images of cut shoots with thresholding of disease using ImageJ and the automated software. Shoots were inoculated with either the virulent strain *Pss* 9097 or with sterile 10 mM MgCl_2_ as a control. **a** Original image, **b** thresholding with Image J, **c** thresholding with automated software. *1*: Cherry cv. Van, *2*: Cherry cv. Napoleon, *3*: Cherry cv. Roundel, *4*: Cherry cv. Merton Glory, *5*: Plum cv. Victoria, *6*: Plum cv. Marjorie’s seedling

To determine the utility of our image analysis software we compared results with both a current method of disease image analysis and expert measurements made by eye. 84 images (block 1 and 2) (e.g. Fig 1a) were analysed by our software to determine percentage area of necrosis, which was then correlated with the output for the same images produced using ImageJ manual thresholding (Fig. 1b). We also used the software to determine the length of necrosis on each shoot (at the longest point), which was correlated with data for the same images, measured manually using a caliper. The software produced both a pictorial output (e.g. Fig 1c) and raw data (available on github).

**Fig. 2:**
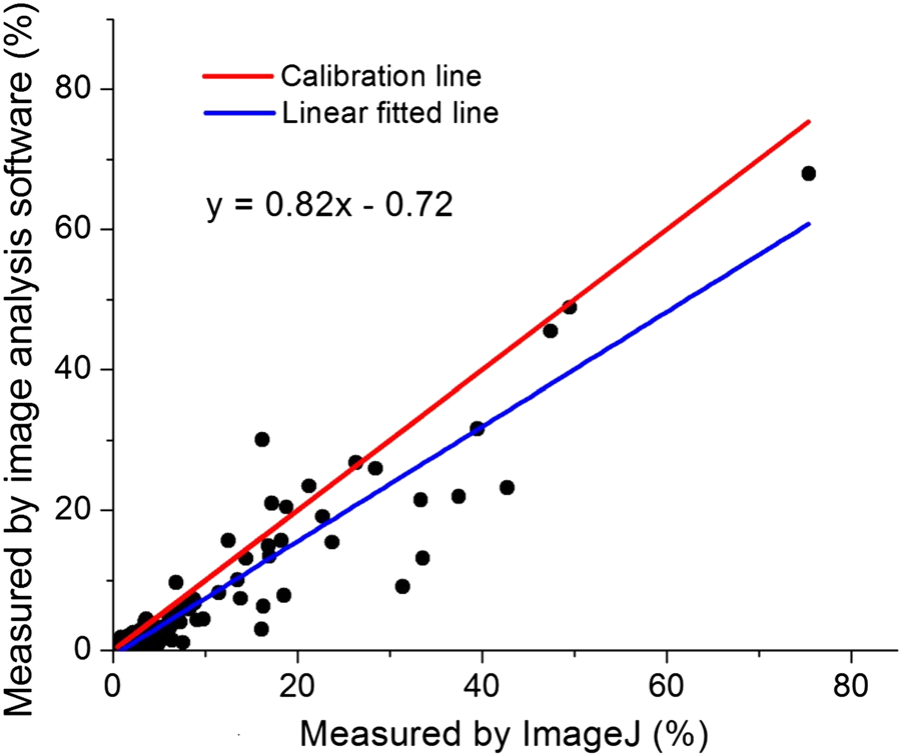
Comparison between the measurements by automated image analysis software and assessment by ImageJ on diseased area. The *linear fitted line* indicated the deviation from the ideal calibration line

**Fig. 3:**
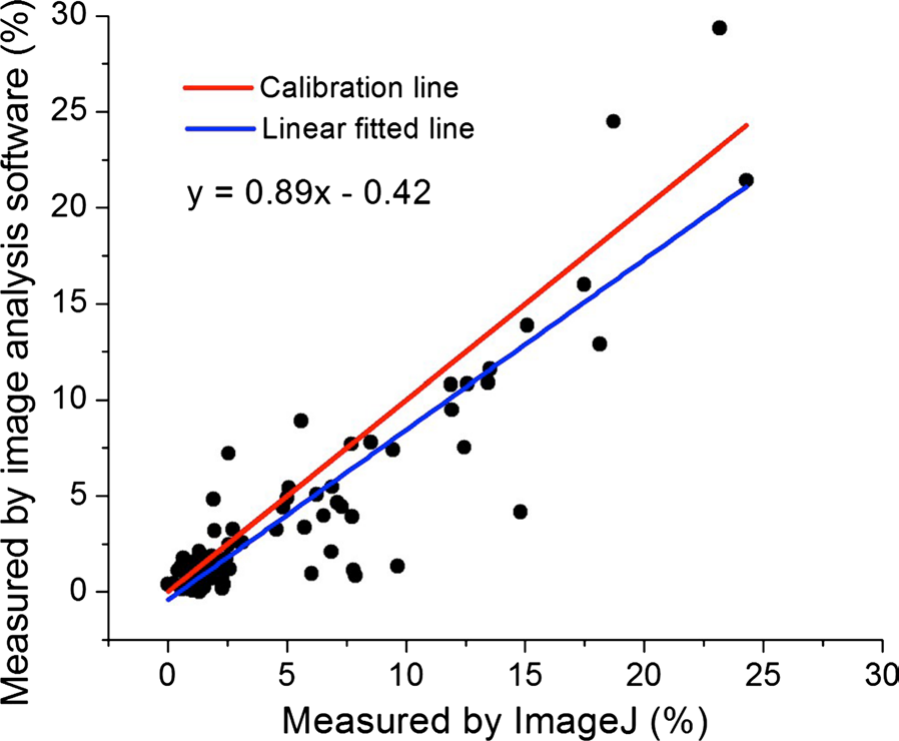
Comparison between the measurements by automated image analysis software and manual measurements by experts on the length of the diseased area. The *linear fitted line* indicated the deviation from the ideal calibration line

Correlation analysis and linear regression indicated results were highly similar using the image analysis software and the other methods of assessment. Figure 2 shows the correlation of percentage area of necrosis whilst Fig. 3 shows the same for necrosis length. A linear regression produced r^2^ values of 0.87 and 0.81 for percentage area and length respectively. In both Figs. 2 and 3, there was deviation between the linear regression line and the ideal calibration line. This difference between methods likely resulted from using an arbitrarily threshold in ImageJ and subjective labelling of diseased pixels in training images. To further test this, Lin’s concordance coefficient [45] was calculated with rhoC values of 0.9 (moderate correlation) and 0.89 (poor correlation as <0.9) for the area and length data respectively. Due to the lower score for the length data when comparing manual measurement and the new software, this data was not used in further analysis of the experiment. This poor rhoC value for the length dataset could be due to manual assessment of length being more subjective. It was sometimes difficult to measure length of necrosis accurately due to natural blemishes on the sample shoots. The automated software could provide a more objective method than classification by eye, however this would need further testing to validate. Overall, the correlation analysis indicated that the automated software could produce results comparable to currently used manual assessment.

The accuracy of the automated measurements relied on an expert’s selection of diseased areas on the images used as the training data. This was necessary to ensure all the typical colours of both diseased and healthy areas were included, reducing the potential for misclassification. The criteria used during prediction of the percentage disease were selected empirically. To our knowledge this is the first time that image analysis and machine-learning algorithms have been applied to disease quantification on plant shoots. Compared with assessment by eye/use of ImageJ manual thresholding, the image analysis software only needs to be trained once by an experienced expert. Many images captured under the same lighting condition can therefore be processed using the same model, which could reduce the subjectivity. The time taken to process all 420 images was approximately 42 s (0.1 s per image) with current hardware and ANN model, so the image analysis software was much faster than traditional methods (ImageJ 60–100 s per sample). The results were also compared with other common image thresholding methods such as fixed thresholding and Otsu’s method. It was found that the fixed thresholding produced a comparable correlation with manual assessment (r^2^ = 0.86) but Otsu’s thresholding methods showed poor results (see Additional file 1: Figure S6 and S7).

With a proper training dataset, the chosen method provided a fast, automated and objective method for disease quantification on cherry shoots. It could be utilised for general disease quantification during other biological experiments with different illumination condition. ANN is a more flexible approach than other thresholding methods, since biologists only need to label regions as diseased or healthy rather than arbitrarily determining a threshold for disease. Further development of the software could involve more input parameters such as texture information, so ANN is more extendable to other input variables.

### Development of automated image analysis software and a graphical user interface

In order to make the software user-friendly, a graphical user interface was developed. The GUI can be used to select the training data on a series of images from a particular folder (see Additional file 1: Figure S1). This selection is semi-automatic as user interaction is necessary to drag the mouse and draw a rectangle within healthy and diseased regions. The colour information of all the pixels inside the rectangle is recorded as healthy or diseased to train the ANN model.

The trained ANN model can subsequently be applied to calculate the percentage area of necrosis. The pixels labelled as diseased are coloured as red (Additional file 1: Figure S2). The resulting image with false colour can be further analysed to estimate the length of disease by measuring the height of the fitted rectangles (Additional file 1: Figure S3). The source code of the software is available on Github (https://github.com/eastmallingresearch/Cherry_shoots).

### Results of pathogenicity assays on cherry and plum

Following training, the automated image analysis software was used for a resistance screen to produce percentage area necrosis data for six strains of *P. syringae* inoculated onto four cultivars of cherry and two cultivars of plum. The strains included *P. syringae* pv. *morsprunorum* race 1 isolated from cherry (5244) and plum (5300), *P. syringae* pv. *morsprunorum* race 2 isolated from cherry (5255) and *P. syringae* pv. *syringae* isolated from cherry (9097) and plum (9293). A strain isolated from hazelnut (*P. syringae* pv. *avellanae*) was also used for comparison as a non-pathogen of *Prunus*.

The plant cultivars (cvs) were chosen as they have a range of susceptibility to the different races of *P. syringae* that infect *Prunus*. The cherry cv Van is reported to be universally susceptible, whilst cv Merton Glory is tolerant/has a lower susceptibility to the pathogen [46, 47]. The cultivars Napoleon and Roundel are reported to show differential susceptibility to the different races of *P. syringae* pv*. morsprunorum* [47], with cv Napoleon being resistant to R2 but susceptible to R1 and vice versa for cv Roundel. For plum, the cv Victoria is highly susceptible, while cv Marjorie’s Seedling is reportedly resistant/tolerant [48].

**Fig. 4:**
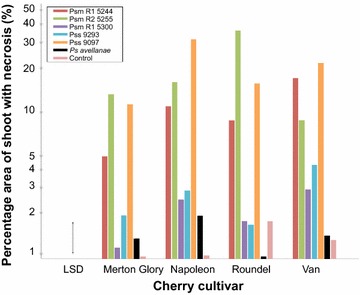
Percentage area of necrosis on cherry shoots inoculated with *P. syringae* for four cherry cultivars (plotted on a log scale with back-transformed values as the scale). Cv Van is universally susceptible, whilst cv Merton Glory has tolerance/lower susceptibility. Cv Napoleon is resistant to *Psm* R2 but susceptible to R1 and vice versa for cv Roundel. The control was sterile 10 mM MgCl_2_. The mean values were calculated using ANOVA. *LSD* Least Significant Difference

**Fig. 5:**
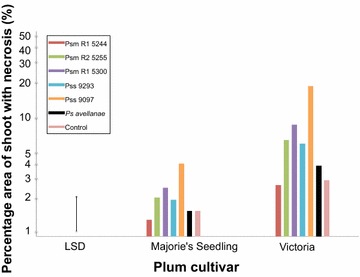
Percentage area of necrosis on plum shoots inoculated with *P. syringae* for two plum cultivars (plotted on a log scale with back-transformed values as the scale). Cv Victoria is highly susceptible and cv Marjorie’s Seedling is resistant/tolerant. The control was sterile 10 mM MgCl_2_. Mean values were calculated using ANOVA. *LSD* Least Significant Difference

The different strains of *P. syringae* caused variable levels of necrosis on shoots of cherry (Fig. 4) and plum (Fig. 5). An analysis of variance (ANOVA) was performed using the log transformed percentage data (Additional file 1: Figure S4). The ANOVA revealed that there was a significant effect of *Pseudomonas* strain on percentage area of necrosis (p < 0.001, df = 6), likely due to variation in the virulence of the different strains. There was no significant difference in percentage area of necrosis between the two *Prunus* species (p = 0.06, df = 1) indicating both species exhibit similar levels of susceptibly to the disease. However there was a significant interaction between *Prunus* species and *P. syringae* strain (p < 0.001, df = 6). This indicates that the different *P. syringae* strains show differential virulence on cherry and plum (Figs. 4, 5).

On cherry, the three strains isolated from cherry (*Psm* R1 5244, *Psm* R2 5255 and *Pss* 9097) were generally associated with severe necrosis (>5 % of total shoot area), whilst necrosis caused by other strains failed to exceed 5 % shoot area. *Pss* 9097 caused significant symptom development on all cultivars, whereas necrosis caused by the two races of *Psm* isolated from cherry, varied considerably between cultivars. This supports previous hypotheses that cherry cultivars exhibit differential susceptibility towards the two races of *Psm* [49]. In the global ANOVA (Table S1) there was no overall interaction between strain, cultivar and species. However, when the comparison was restricted to Van and Roundel, a highly significant interaction (p = 0.004) was detected between the two cultivars and the strains, which is driven by the differences between *Psm* R1 and *Psm* R2. The cultivars Roundel and Van showed differential susceptibility to the two *Psm* races. On Van, *Psm* R1 caused more severe necrosis than *Psm* R2, whilst on Roundel this response was reversed. One reason for this could be that plant immunity responses to the different races vary between cultivars. Overall the results indicated that no single cultivar of cherry was tolerant to all strains. The symptoms on Merton Glory never exceeded 25 % of the shoot area, indicative of partial tolerance. Therefore, a cross between Merton Glory and a more susceptible cultivar could be used to further investigate the genes involved in tolerance/resistance.

On plum (Fig. 5), the level of necrosis was generally higher on cv Victoria compared to Marjorie’s Seedling. Interestingly, the two strains originally isolated from plum (*Psm* R1 5300 and *Pss* 9293) caused a higher level of necrosis on plum than on cherry. Also, when inoculated on plum they generally caused more severe necrosis than strains isolated from cherry and hazelnut (*Psm* R1 5244, *Psm* R2 5255 and *Ps. avellanae*). The virulence of these plum strains on plum could be due to host-specific factors, which allow the pathogens to survive longer and cause more necrosis in their natural (homologous) host.

The plum cultivar Marjorie’s Seedling showed some resistance to most strains, with the severity of necrosis being similar to the control (inoculation with sterile MgCl_2_). It was also more tolerant to the virulent *Pss* strain 9097. This supports previous reports that this cultivar is tolerant to bacterial canker. Therefore, Marjorie’s Seedling could be a target for further investigations of the genetics of resistance.

## Conclusion

In this study a method for automated image analysis to measure the severity of disease symptoms was developed using a machine learning approach. To validate the reliability of our automated software, cherry and plum shoot images were analysed to measure necrosis using the free program ImageJ [31]. The ImageJ analysis was based on the hue value of the colour images and the threshold between the diseased and healthy area was determined arbitrarily, resulting in a loss of the colour information from the other two channels. The 3D shape of cherry shoots resulted in shadows, leading to a colour similar to the diseased area in grayscale images or the hue channel of HSV space, but still distinguishable by the naked eye. Furthermore, manual image analysis using ImageJ can only process one image at a time and the images need to be loaded manually before applying the thresholding technique, which is extremely time consuming.

Due to the variation in the colour of diseased and healthy areas, it is difficult to set arbitrary thresholds for all three channels of colour space. The new image analysis method employed artificial neural networks (ANN) for the training and classification of a colour dataset. With the expert’s selection of training data featured by the RGB values and ANN as the classification algorithm, the quantification of disease was highly correlated with a subjective quantification method implemented in ImageJ. The software greatly reduced the time requirements for disease assessment when compared to manual thresholding with imageJ. This assisted in the objective identification of differences in cultivar susceptibility to the various strains that cause bacterial canker. This software therefore provides opportunities to shorten time taken for disease assessment dramatically. The software would facilitate the use of the cut shoot test for high-throughput screening during breeding programmes. This would enable the selection of putatively resistant material from mapping populations, which often contain hundreds of individuals. Finally, this software is highly adaptable and could be implemented during the screening of other tree diseases.

## Methods

### Bacterial strains

Strains of *Pseudomonas syringae* were grown on King’s B agar (Sigma) at 25 °C. For liquid culture, strains were grown in Luria Broth (Melford) at 25 °C, 150 rpm. Strains were obtained from various sources (Table 1) and included representatives of the three major clades that infect *Prunus* (*Psm* Race 1, *Psm* Race 2 and *Pss*) as well as an out-group strain belonging to pv. *avellanae* which was isolated from hazelnut (*Corylus avellana*).

**Table 1:** List of bacterial strains used in pathogenicity assays, with source host and reference

Strain	Species	Pathovar	Race	Source of isolation	Isolate curator
5244	*P. syringae*	*morsprunorum*	1	*Prunus avium*	SJ Roberts
5300	*P. syringae*	*morsprunorum*	1	*Prunus domestica*	SJ Roberts
5255	*P. syringae*	*morsprunorum*	2	*Prunus avium*	SJ Roberts
9097	*P. syringae*	*syringae*	–	*Prunus avium*	SJ Roberts
9293	*P. syringae*	*syringae*	–	*Prunus domestica*	SJ Roberts
BPIC631	*P. syringae*	*avellanae*	–	*Corylus avellana*	DS Guttman

### Plant material

Dormant first-year shoots were collected from mature cherry and plum trees in December 2014 at East Malling Research, Kent.

### Pathogenicity assay on cut shoots

The cut shoot pathogenicity assay was performed as in previous studies [3, 8]. Each cultivar x strain treatment was replicated 10 times, resulting in 420 inoculations. To prepare the bacteria, single colonies were inoculated in LB and shaken overnight. These cultures were spun down using a centrifuge (4000 rpm, 10 min) and re-suspended in 10 mM MgCl_2_. The concentration was adjusted to 1 × 10^7^ CFU/ml (confirmed by dilution plating) and sterile 10 mM MgCl_2_ was used for the control. For the plant material, dormant first-year shoots of similar diameter (5 mm) were collected from cherry and plum trees in December and cut into 10 cm sections using secateurs. These were surface-sterilised in 0.5 % hypochlorite for 5 min and rinsed with tap water. The shoot sections were air-dried overnight.

To inoculate, the top 5 mm of each shoot tip was removed with a scalpel and dipped for 5 min in the bacterial suspension. The wound was covered with parafilm (Fisher Scientific, UK) and the shoot bases were freshly cut (approx. 5 mm) and placed in transparent-boxes immersed in water to a depth of 20 mm. The shoots were incubated in the closed boxes at 15 °C with 16-hour light, 8-hour dark cycle for 1 week. Separate boxes were used for each bacterial isolate to prevent cross-contamination. Next, the shoots were transferred to −2 °C for one week to simulate frost damage. Finally, the basal 10 mm of each shoot was removed and they were placed in a completely randomised design (generated using Genstat [50]) in water-soaked Oasis Foam (Oasis Floral, UK) in trays containing 30 mm of water. These were incubated for a further 4 weeks at 15 °C with the same light conditions as previously described. The trays were covered with cling-film to maintain a high humidity.

The shoots were assessed for severity of stem canker by peeling back the uppermost layer of bark from the top 30 mm of the shoot to expose the symptoms, which were photographed digitally. The length of necrosis was also manually measured with a caliper.

### Imaging system

All the images were captured using a SLR camera (Canon EOS 1000D) with 53 mm focal length and 1/15 s exposure time. Two 60 W incandescent light bulbs were used to illuminate the samples from each side. The distance between the lens and the samples was 35 cm. Due to the high resolution of the imagery device (3888 × 2592 pixels), three shoots were placed on a spectralon white platform (SphereOptics) and imaged together in order to enhance the contrast between the foreground and background. The images were captured using EOS utility software (Canon) and saved as JPG files. Individual shoots were cropped from each image and due to small variations in the size of shoots, the resolution of the images varied from 43 × 754 to 282 × 839 pixels. All the images were saved and processed on a Dell desktop computer (Intel^®^ Xeon(R) CPU X5560 @ 2.80 GHz × 16). The automated image analysis software was written in C++ [51] utilising the OpenCV Library [52] on an Ubuntu 14.04 operating system.

### Statistics

Genstat [50] was used to perform the statistical analysis using a nested ANOVA (nesting cultivar by species), whilst Excel [53] was used to produce bar charts (Fig. 4 and 5). The residuals of ANOVA tests were assessed for normality using qqnorm (residuals). If the residuals were not normally distributed the data was log transformed (with the addition of 0.1 to area prior to log transformation) and the ANOVA repeated. Log transformation was selected rather than the more conventional square root transformation, as the full spectrum of percentage disease was not used and the relationship between residuals and fitted values was less biased. Furthermore, a log transformation is more appropriate to study multiplicative interactions between factors. Full ANOVA tables and residual plots can be found in the supplementary information (Additional file 1: Tables S1, S2; Figures S4, S5). A complete randomised design for the positioning of cherry shoots in trays after inoculation was produced using Genstat [50].

### Image analysis with ImageJ

ImageJ [31] was used to manually measure the disease severity on an image-by-image basis. Firstly, the three cherry shoots were cropped from the original image and converted from RGB to HSI colour space. A threshold was manually chosen to determine the total number of pixels in the shoot (compared to the total in the whole image containing the background). The total number of pixels in the shoot was named R1. The second threshold on the hue channel was used to segment the diseased and healthy areas. As the diseased area always showed darker intensity than the healthy area, the background could be easily separated. The total number of pixels in the diseased area was called R2. The proportion of the diseased area was calculated using the ratio of the diseased area (R2) to the total shoot area (R1).

### Automated image analysis software

The automated image analysis software was developed in C++ with open computer vision library (OpenCV 2.4.9), and the interface was designed by Qt designer. The software is programmed to load all images in a single folder and process them in a batch with the prediction parameters included and output the percentage area of necrosis and the necrosis length.

The original images were converted to grayscale and the pixels belonging to the three shoots were segmented from the background by setting an arbitrary threshold. All the contours were detected, those <500 pixels were considered noise and were discarded leaving only the three shoots. Rectangles were fitted to all three contours, which were cropped from background and saved as three individual images for further processing.

A feed-forward artificial neural network (ANN) was implemented for the imaging classification. The ANN model consists of one input layer with three neurons, one binary output layer and one hidden layer with 16 neurons (Additional file 1: Figure S8). The input layer was the same size as the sample feature variables (Red Green and Blue) in this experiment. The input is passed to each neuron of the hidden layer and summed up with certain weights. A symmetrical sigmoid function was applied to the sum for each neuron and the output of each neuron on the hidden layer was further summed up with weights to the output. The model is trained with a training dataset to adjust the weights iteratively in order to minimize the error between ideal and real output.

Thirteen images were selected for extraction of the training dataset. The expert labelled pixels as diseased by drawing squares of different sizes by pressing the left mouse button to the diseased region. Similarly, the right button was used to label pixels as healthy. The original images were kept in RGB format and the R, G and B values were used as the three variables for the training phase.

In the prediction phase, the segmentation was applied first with an arbitrary threshold to separate the pixels belonging to the shoot from the background and input to the classification model, which reduces the computation cost. The R, G and B values for each pixel were taken as feature variables, classified by ANN and labelled as diseased or healthy. The pixels labelled as diseased were also false coloured as red for visualization. Ratios between the number of pixels in the diseased area and total area were calculated automatically and saved in text files. The length of necrosis measurement was based on the false colour image. Any red regions with less than 10 pixels were regarded as noise so were removed, whilst all other red regions were fitted with rectangles. If the area of fitted rectangles were less than 10,000 pixels, the correspondent red regions were further removed unless the regions were near the top of the shoots (at the point of infection). This was required to remove any blemishes that were not due to the disease. The final length was calculated by measuring the difference between the top and bottom of the rectangle.

The software is available from the East Malling github repository, (www.github.com/organizations/eastmallingresearch/).

## Additional file

10.1186/s13007-015-0100-8 A standard view of the GUI during manual selection of training data. **Figure S2.** A standard view of the GUI during percentage area of necrosis estimation. **Figure S3.** A standard view of the GUI during necrosis length estimation. **Figure S4.** Visual output of ANOVA from Genstat including the histogram of residuals, fitted-value plot and normality plots. **Figure S5.** Interaction plot showing percentage necrosis for all *P. syringae* strains on the different *Prunus* species cherry and plum. The mean values from the ANOVA were used and Least Significant Difference bars calculated during the ANOVA. The means are plotted on a log scale with back-transformed values as the scale. Strains isolated from cherry are highlighted in red whilst those isolated from plum are highlighted in purple. **Figure S6.** The relationship between the percentage area of necrosis measured using ImageJ or automated image analysis software using fixed thresholding. The linear fitted line indicated the deviation from the ideal calibration line. **Figure S7.** The relationship between the percentage area of necrosis measured using ImageJ or automated image analysis software using adaptive thresholding. The linear fitted line indicated the deviation from the ideal calibration line. **Figure S8.** Graphical depiction of an artificial neural network (ANN) model containing one input layer with three neurons, one binary output layer and one hidden layer with sixteen neurons. **Table S1.** List of bacterial strains used in pathogenicity assays, with source host and reference. **Table S2.** ANOVA table generated using Genstat. The ANOVA was performed on the log transformed (+0.1) raw data values. The formula used was aov(strain*(species/cv)+box).
