# Supplementary material for: Rapid, automated detection of stem canker symptoms in woody perennials using artificial neural network analysis
Source: Plant Methods. 2015 Dec 24;11:57. doi: 10.1186/s13007-015-0100-8 (PMC4690310; doi:10.1186/s13007-015-0100-8)
Supplement: Supplementary file 1 — 10.1186/s13007-015-0100-8 A standard view of the GUI during manual selection of training data. Figure S2. A standard view of the GUI during percentage area of necrosis estimation. Figure S3. A standard view of the GUI during necrosis length estimation. Figure S4. Visual output of ANOVA from Genstat including the histogram of residuals, fitted-value plot and normality plots. Figure S5. Interaction plot showing percentage necrosis for all P. syringae strains on the different Prunus species cherry and plum. The mean values from the ANOVA were used and Least Significant Difference bars calculated during the ANOVA. The means are plotted on a log scale with back-transformed values as the scale. Strains isolated from cherry are highlighted in red whilst those isolated from plum are highlighted in purple. Figure S6. The relationship between the percentage area of necrosis measured using ImageJ or automated image analysis software using fixed thresholding. The linear fitted line indicated the deviation from the ideal calibration line. Figure S7. The relationship between the percentage area of necrosis measured using ImageJ or automated image analysis software using adaptive thresholding. The linear fitted line indicated the deviation from the ideal calibration line. Figure S8. Graphical depiction of an artificial neural network (ANN) model containing one input layer with three neurons, one binary output layer and one hidden layer with sixteen neurons. Table S1. List of bacterial strains used in pathogenicity assays, with source host and reference. Table S2. ANOVA table generated using Genstat. The ANOVA was performed on the log transformed (+0.1) raw data values. The formula used was aov(strain*(species/cv)+box). [file 13007_2015_100_MOESM1_ESM.pdf]

Figure S1. A standard view of the GUI during manual selection of training data

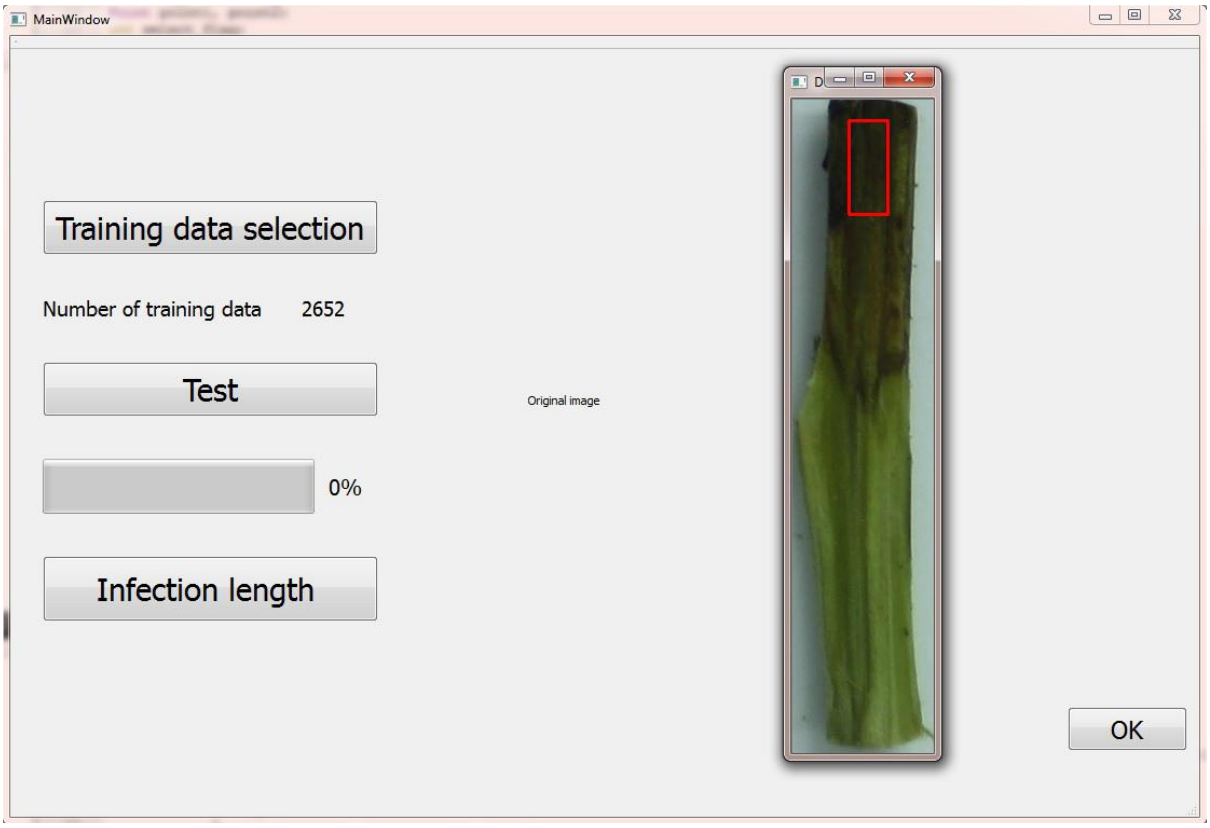

Figure S2. A standard view of the GUI during percentage area of necrosis estimation

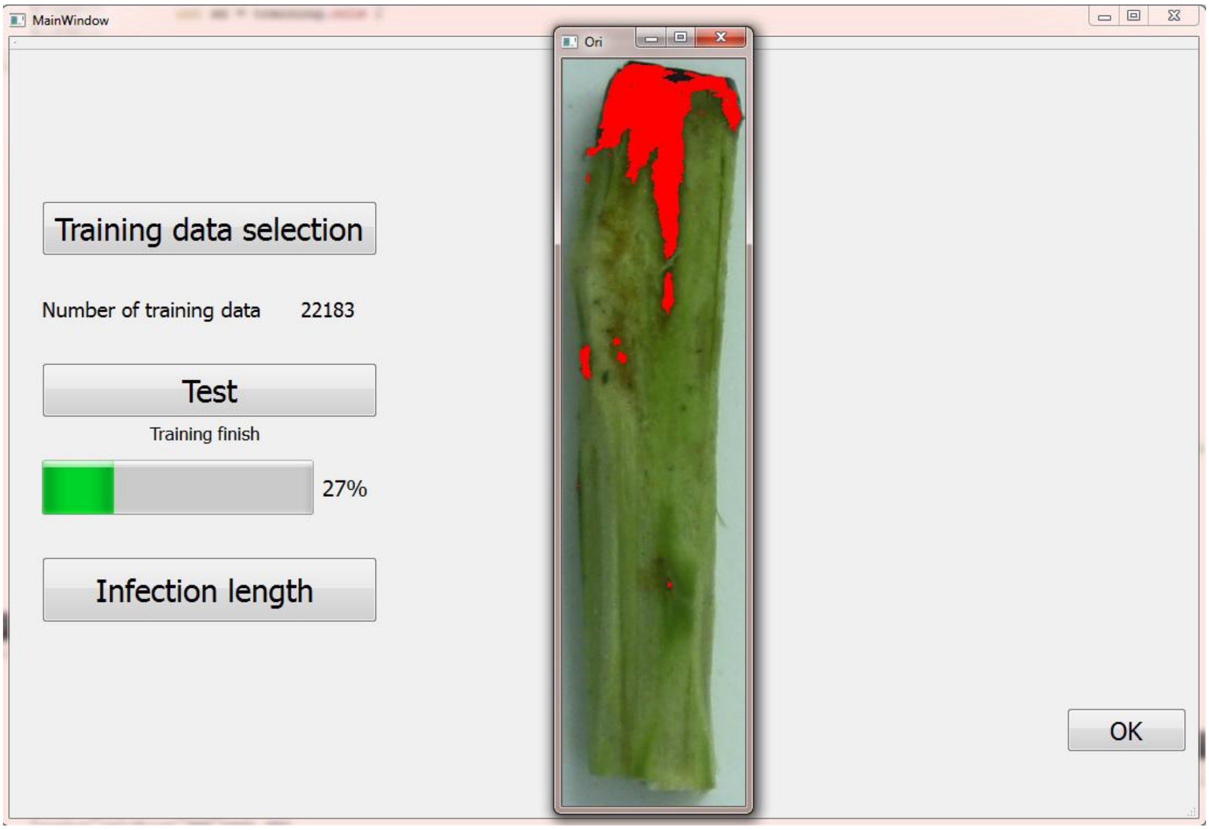

Figure S3. A standard view of the GUI during necrosis length estimation

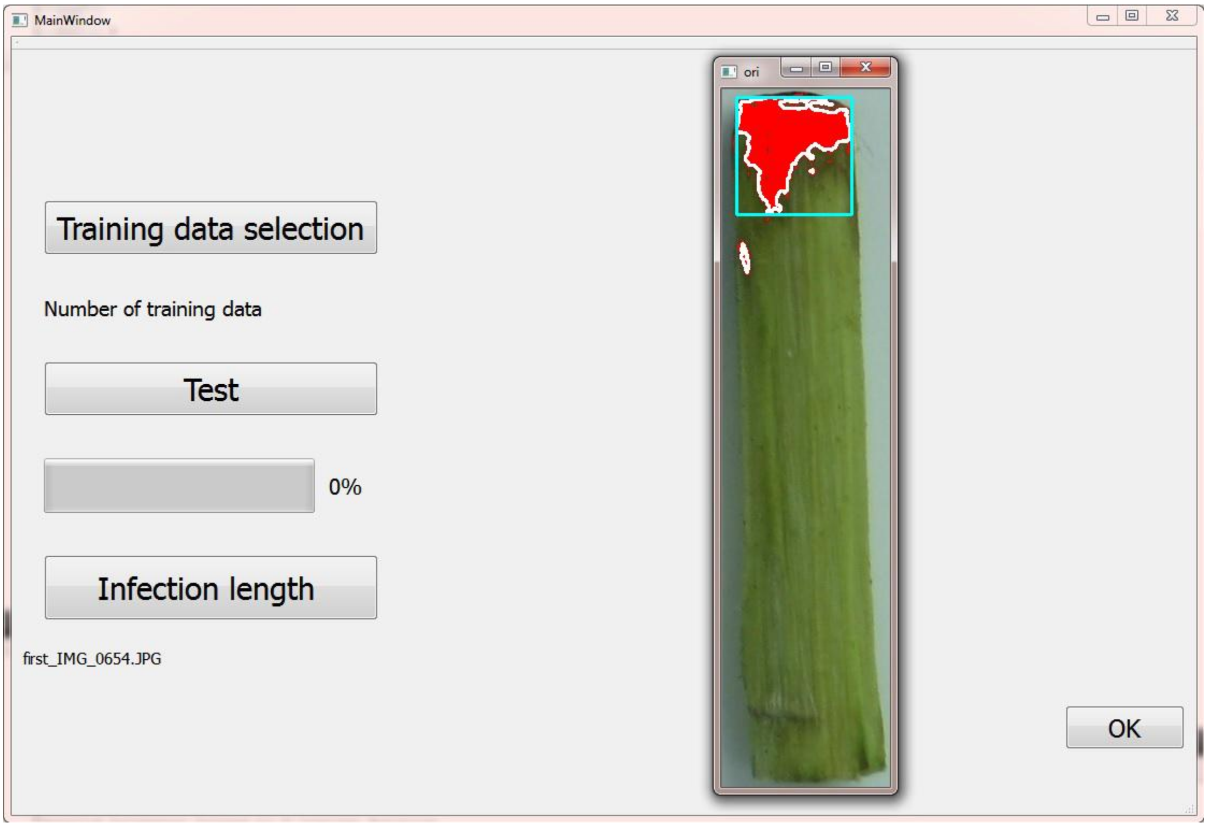

Figure S4. Visual output of ANOVA from Genstat including the histogram of residuals, fitted-value plot and normality plots.

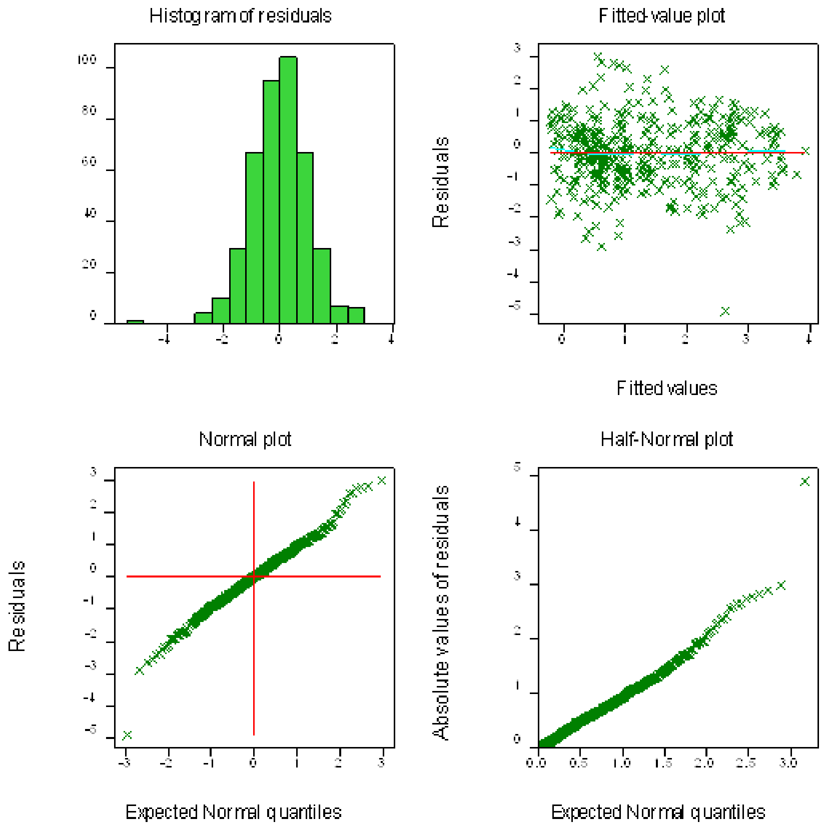

Figure S5. Interaction plot showing percentage necrosis for all *P. syringae* strains on the different *Prunus* species cherry and plum. The mean values from the ANOVA were used and Least Significant Difference bars calculated during the ANOVA. The means are plotted on a log scale with back-transformed values as the scale. Strains isolated from cherry are highlighted in red whilst those isolated from plum are highlighted in purple.

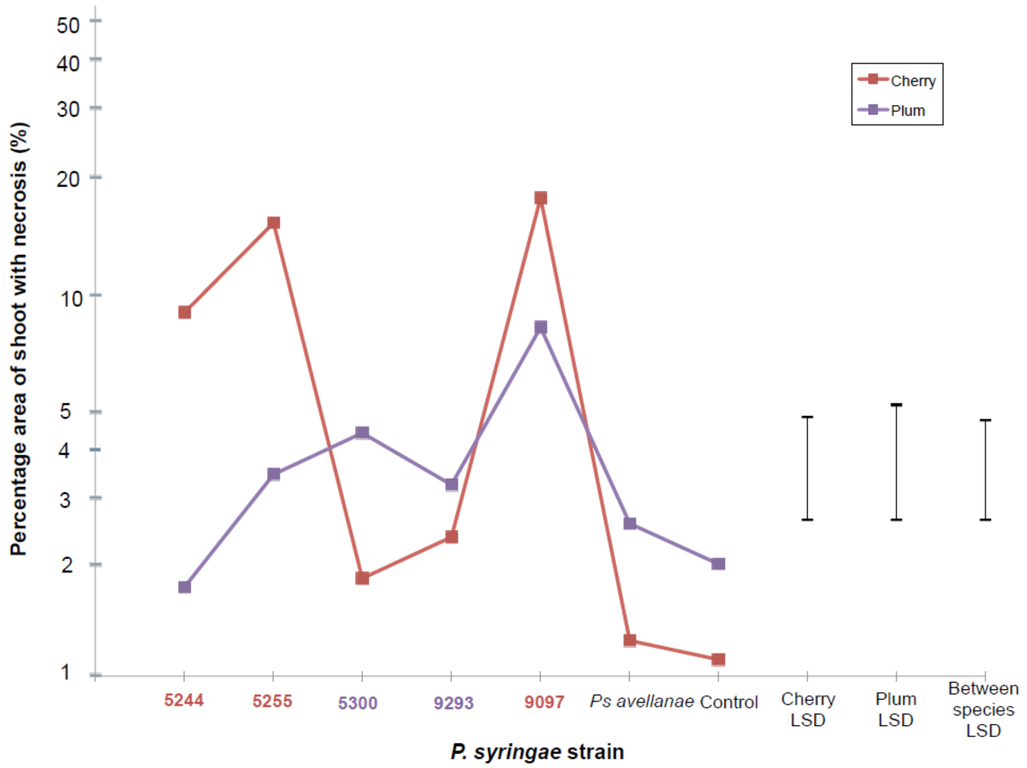

Figure S6. The relationship between the percentage area of necrosis measured using ImageJ or automated image analysis software using fixed thresholding. The linear fitted line indicated the deviation from the ideal calibration line.

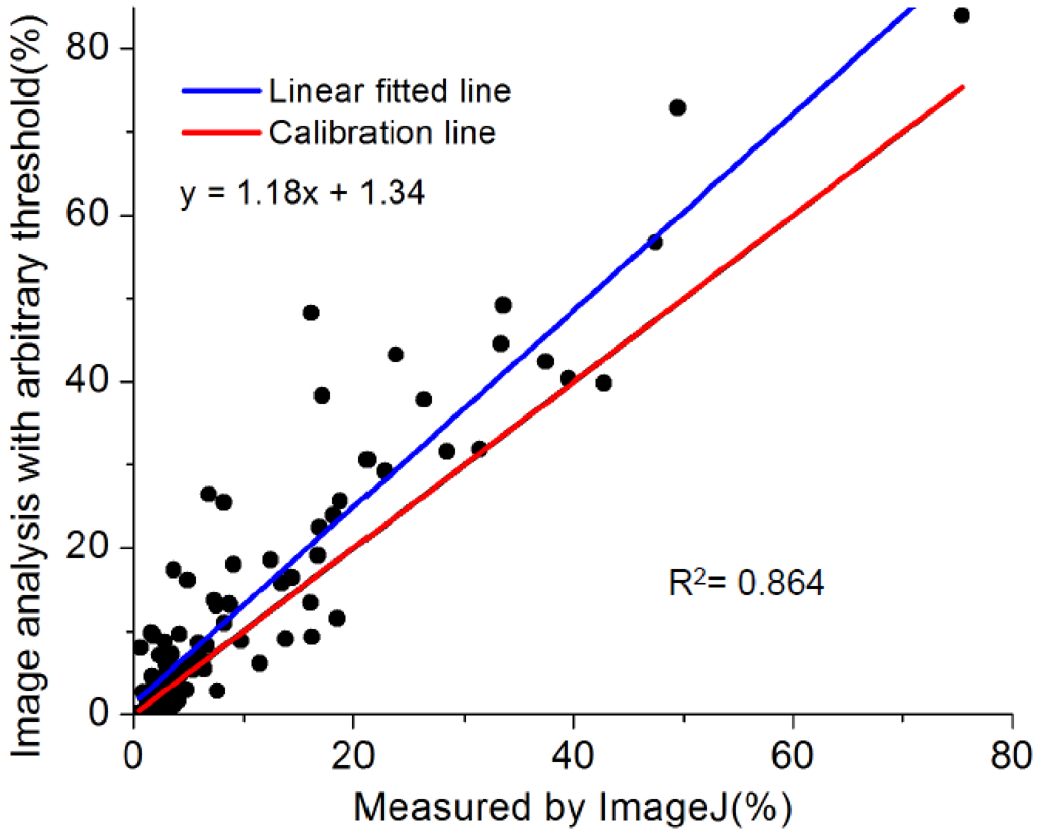

Figure S7. The relationship between the percentage area of necrosis measured using ImageJ or automated image analysis software using adaptive thresholding. The linear fitted line indicated the deviation from the ideal calibration line.

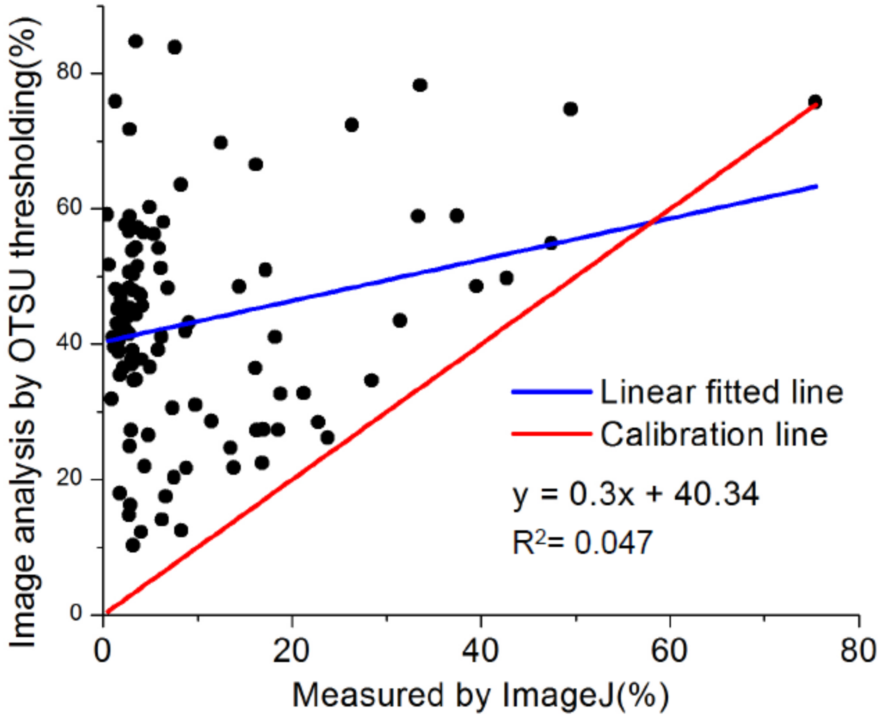

Figure S8. Graphical depiction of an artificial neural network (ANN) model containing one input layer with three neurons, one binary output layer and one hidden layer with sixteen neurons

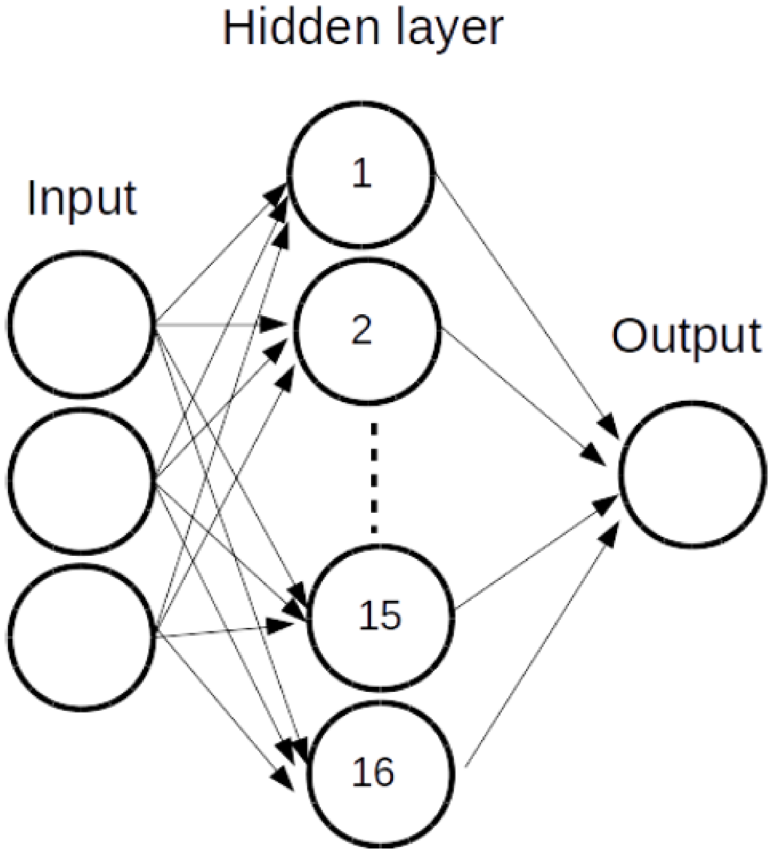

Table S1. ANOVA table generated using Genstat. The ANOVA was performed on the log transformed (+0.1) raw data values. The formula used was aov(strain*(species/cv)+box)

Source of variation	d.f.	(m.v.)	s.s.	m.s.	v.r.	F pr.
**box stratum**	9		8.454	0.939	0.82	
**box.*Units* stratum**						
**Species**	1		3.895	3.895	3.39	0.066
**Strain**	6		267.715	44.619	38.82	<.001
**species.cv**	4		46.591	11.648	10.13	<.001
**species.strain**	6		87.822	14.637	12.73	<.001
**species.cv.strain**	24		30.332	1.264	1.1	0.341
**Residual**	368	1	422.974	1.149		
**Total**	418	1	867.032			

ANOVA FORMULA: strain*(species/cv)+box

Table S2. Table of means and Least Significant Difference values generated from the ANOVA.

Tables of means from ANOVA

Variate: LOG((area+0.1))

Grand mean 1.342

**species**	**cherry**	**plum**
	1.41	1.206
**rep.**	280	140

		**strain**						
**species**	**cv**	**5244**	**5255**	**5300**	**9293**	**9097**	***Ps avellanae***	**Control**
**cherry**	mgl	1.566	2.541	0.138	0.641	2.383	0.281	-0.08
	nap	2.352	2.727	0.896	1.033	3.399	0.638	0.017
	round	2.129	3.539	0.557	0.501	2.706	-0.057	0.549
	van	2.792	2.132	1.052	1.433	3.029	0.326	0.258
**plum**	mseed	0.273	0.702	0.893	0.658	1.366	0.626	0.449
	vict	0.946	1.832	2.125	1.755	2.88	1.333	1.04

Least significant differences of means (5% level)

**Table**	**species**	**strain**	**species cv**	**species strain**	**species cv strain**
**rep.**	**unequal**	60	70	**unequal**	10
**d.f.**	368	368	368	368	368
**l.s.d.**	0.2182	0.3849	0.3564	0.6667 **min.rep**	0.9428
				0.5774 **max-min**	
				0.4714 **max.rep**	
